# Toxicological Effects of Silver-Modified Bentonite Nanocomposites on Microalgae: Impact on Cell Growth, Antioxidant Enzymes, and Gene Expression

**DOI:** 10.3390/nano15080629

**Published:** 2025-04-20

**Authors:** Oumayma Ghariani, Jihen Elleuch, Anna Maria Ferretti, Stefano Econdi, Chiara Bisio, Philippe Michaud, Imen Fendri, Matteo Guidotti, Slim Abdelkafi

**Affiliations:** 1 Enzymatic Engineering and Microbiology Laboratory, Algae Biotechnology Unit, National Engineering School of Sfax, University of Sfax, Sfax 3038, Tunisia; oumayma.ghariani@enis.tn (O.G.); jihen.elleuch@enis.tn (J.E.); slim.abdelkafi@enis.tn (S.A.); 2 CNR-SCITEC Istituto di Scienze e Tecnologie Chimiche “G. Natta”, Via C. Golgi 19, 20133 Milano, Italy; stefano.econdi@scitec.cnr.it (S.E.); matteo.guidotti@scitec.cnr.it (M.G.); 3 CNR-SCITEC Istituto di Scienze e Tecnologie Chimiche “G. Natta”, Via G. Fantoli 16/15, 20138 Milano, Italy; anna.ferretti@scitec.cnr.it; 4 Department of Science and Technological Innovation, DISIT, University of Eastern Piedmont, Via T. Michel 11, 15121 Alessandria, Italy; chiara.bisio@uniupo.it; 5 Université Clermont Auvergne, Clermont Auvergne INP, CNRS, Institut Pascal, F-63000 Clermont-Ferrand, France; 6 Laboratory of Plant Biotechnology Applied to Crop Improvement, Faculty of Science of Sfax, University of Sfax, Sfax 3029, Tunisia; imen.fendri@fss.usf.tn

**Keywords:** bentonite, montmorillonite, silver-exchanged clays, metal nanoparticles, Chlorophycae, *Chlamydomonas* sp., qPCR, gene expression

## Abstract

The increasing use of nanostructured silver-containing inorganic materials raises concerns about their impact on aquatic organisms. This study assessed the toxicity of silver-modified bentonite composites on *Chlamydomonas* sp. Two materials were tested: silver-exchanged bentonite (Ben-Ag) and its reduced form (Ben-Ag (H_2_)).Microalgae were exposed to 0.5 IC_50_, 1.5 IC_50_, and 2 IC_50_. Ben-Ag showed higher toxicity than Ben-Ag (H_2_), which even promoted algal growth at low doses. Fluorescence microscopy revealed morphological shrinkage in treated cells. Increased phenol content, elevated malondialdehyde (MDA) levels, and altered antioxidant enzyme activities further confirmed Ben-Ag toxicity, along with reduced growth and photosynthetic pigments. Transcriptomic analysis revealed significant changes in gene expression under Ben-Ag exposure. Genes involved in photosynthesis (*petB*, *psbL*), caspase activity (*casp*), and carotenoid metabolism (*Q2CHY*) were down-regulated, indicating stress-induced damage. In contrast, genes encoding stress response enzymes (*SOD*, *peroxidase*), carbon metabolism enzymes (*rbcL*, *PGQ1*), and β-carotene biosynthesis (*Q2BKT*) were up-regulated, reflecting cellular defense mechanisms. Overall, the study highlights the high toxicity of Ben-Ag to *Chlamydomonas* sp., emphasizing the importance of evaluating environmental risks before using such materials in aquatic environments.

## 1. Introduction

Due to its immense potential and practical implications, nanotechnology is currently considered one of the most appealing research fields in a number of nations [1]. In many branches of science, medicine, agriculture, technology, and industry, nanotechnology refers to the study, synthesis, manufacturing, and processing of materials and structures at the nanoscale [2]. Many nanostructured materials are being developed to fulfill a variety of human needs. Due to their numerous potential benefits, applying nanotechnology to food, textile, and biomedical applications is essential in today’s cutthroat market [3]. As nanostructured materials are used more frequently, their unintentional introduction into the environment is inevitably increased, which could have negative health effects on aquatic life. For this reason, the toxicity data of these solids are crucial for the thorough assessment of any possible risks to environmental health in aquatic environments [4].

Metal ions such as silver, copper, and zinc are known to have strong inhibitory and bactericidal effects against a wide range of bacteria [5]. Particularly, researchers are primarily interested in silver (Ag) nanoparticles due to their optical, catalytic, and antibacterial properties. However, because of their poor colloidal stability, free Ag nanoparticles present challenges for industrial use [6]. The toxicity of Ag ions to various aquatic organisms, such as algae, fish, and planktonic species, has been reported [7]. Several studies have demonstrated that Ag^+^ ions exhibit toxicity toward aquatic organisms, particularly when they are originated from free nanosized silver particles [8]. The toxicity of Ag+ to microorganisms is attributed to the inactivation of cellular proteins and thiol groups, leading to disruptions in ATP synthesis, ion transport, DNA replication, and respiration processes [8]. To mitigate the adverse effects, an alternative approach involves immobilizing Ag nanoparticles on the surface of inorganic supports, such as clays. The spaces between the lamellae in clay materials are widely accessible, and their intercalation chemistry has been extensively studied [9]. Due to their large surface area, remarkable cation exchange capacity (CEC), and peculiar physical–chemical properties, clays can be used as low-cost and environmentally friendly cleaning agents for contaminants in aqueous phases [10]. As support materials for Ag and ZnO nanoparticles, their adsorption properties increase the efficacy of the distributed nanoparticles by drawing impurities into the open pores [11].

Mineral bentonite is composed of microcrystalline montmorillonite (MMT) particles belonging to a 2:1 phyllosilicate clay, which has octahedral layers sandwiched between two tetrahedral layers. The replacement of exchangeable inorganic cations (e.g., Na^+^, Ca^2+^, H^+^, K^+^) on the inner and outer surfaces of bentonite with inorganic cations such as Ag(I) or Cu(II) species modifies and tunes the chemical properties of the pristine clays for several practical applications [12].

Some nanoparticles are more toxic than their bulk counterparts due to their extremely high surface-to-volume (S/V) ratio, which makes them highly reactive. Their reactivity influences interactions with other chemicals and affects the physiological conditions of various bodily tissues [13]. The potential effects of nanomaterials on aquatic organisms may stem from their non-internalized attachment to the cell surface, which results in a reduction of available light and a shading effect. Furthermore, nanoparticles may penetrate cells and cause the generation of intracellular reactive oxygen species (ROS), which can cause oxidative damage to organic macromolecules, like proteins and DNA, and have an impact on cellular functions [14,15]. Different aquatic species exhibited various degrees of sensitivity to nanomaterials, of which algae were the most sensitive group of organisms, followed by fish, crabs, and bacteria [16]. Taking into account that microalgae are among the most vulnerable organisms to nanomaterials, it is important to note that the harmful effects of nanomaterials on algal cells may be linked to nutrient depletion, the shading effect (which prevents photosynthesis and absorption of light), and direct exposure [17]. Microalgae, as key contributors to aquatic primary productivity, are widely used as model organisms for assessing the ecotoxicity of heavy metals, toxicants, and nanomaterials [6,18,19,20]. In particular, the unicellular green alga *Chlamydomonas reinhardtii*, whose genome has been sequenced, has been used in several studies as a model organism to investigate copper toxicity. It is also important to remember that different microalgae species have differing levels of toxicity in the presence of nanomaterials [21,22].

The aim of this work is to prepare silver-containing clay materials, obtained by inserting well-dispersed Ag sites into bentonite via two different pathways (insertion of Ag^+^ species through cationic exchange and through cationic exchange followed by reduction under hydrogen atmosphere), and investigate the biocidal potential of these two solids. The materials were characterized using Fourier transform infrared (FTIR) and transmission electron microscopy (TEM). Furthermore, their cytotoxicity toward *Chlamydomonas* sp. was assessed by [3-[4,5-dimethylthiazol-2-yl]2,5-diphenyltetrazoliumbromide (MTT) assay. We have studied the toxicity mechanisms of the silver–bentonite nanomaterials at multiple biological levels, including physiological, biochemical, and molecular approaches.

## 2. Materials and Methods

### 2.1. Preparation of Bentonite Clay–AgNO_3_ Powder

Bentonite Globalfeed AR (Ben) was obtained from Laviosa Chimica Mineraria SpA (Livorno, Italy), Silver nitrate (AgNO_3_) (99%) was purchased from Sigma-Aldrich, Germany. Ag^+^-modified bentonite AR (Ben-Ag) was prepared by a cation exchange process. First, 3.74 g of AgNO_3_ was stirred into 60 mL of Milli-Q water to which 3 mL of 28% NH_3_ solution was added. Then, 10 g of bentonite was added over 30 min. The suspension was stirred overnight at room temperature using a magnetic stirrer (400 rpm). At the end of the reaction, the suspension was filtered and the filtrate was washed several times. Then, the collected powder was dried in an oven at 110 °C for 5 h. The obtained powder was named Ben-Ag. Ag (I) gravimetric tests were performed as follows on the residual ion exchange solution to determine the actual Ag(I) content in the sample.

### 2.2. Gravimetric Quantification of Ag(I) by Precipitation

The presence of Ag (I) ions was confirmed through the formation of a white precipitate of AgCl when NaCl was introduced, as indicated by the following reaction:

AgNO_3_ + NaCl → AgCl + NaNO_3_

Here, 14.95 mL of ion exchange washing solution, containing approximately 0.4 g of Ag (I), was transferred into a beaker containing 62 mL of deionized water, weakly acidified with HNO_3_aq. Subsequently, 460 µL of dilute HCl was added in slight excess to ensure complete precipitation of Ag, followed by heating to boiling. Afterward, the solution was allowed to cool in darkness and then filtered through a Gooch funnel, with the precipitate (AgCl) being washed with water acidified with HNO_3_ and then with demineralized water. The resulting precipitate was dried directly on filter paper in an oven at 110 °C, cooled in a desiccator, and finally weighed to determine the Ag (I) content in the sample.

### 2.3. Preparation of Bentonite with Ag Treated with Gaseous Hydrogen

The Ag-containing bentonite (Ben-Ag) was first treated under heat at 200 °C for 1 h (heating rate 10 °C/min) to remove any sorbed water and/or dioxygen. After cooling, the powder was then treated under pure H_2_ at 200 °C for 1 h (heating rate 10 °C/min) to complete the reduction of Ag^+^ species into Ag^0^. The obtained dark gray powder was named Ben-Ag (H_2_).

### 2.4. Characterization

The samples were characterized by DLS using a Zeta Sizer Nanoseries (Malvern, UK). The morphological characterization was performed by a Zeiss Libra 200 FE high-resolution transmission electron microscope (HR-TEM), Carl Zeiss, Toronto, Canada, and the nanocompositional analysis was performed by Oxford X-Stream 2 energy diffused X-ray (EDX) spectroscopy (Oxford Instrumentrs, Abingdon-on-Thames, UK) and Integrated Calibration and Application Tool (INCA) software (ETAS INCA, Stuttgart, Germany). The samples for the TEM and STEM-EDX analysis were dropped, after sonication in isopropanol, on lacey carbon Cu grid 300 mesh and then dried overnight. The elemental composition of the samples was determined using a Perkin Elmer Model 2400 Series II CHNS/O Analyzer (Shelton, CT, USA) with calibration standards (C% = 1, H% = 5, N% = 0.5). FTIR characterization of the powders was performed using a Thermo Fisher Scientific Instrument Nicolet IS 50 FTIR spectrometer (Waltham, Massachusetts, USA), using attenuated total reflection (ATR) in the wavelength range 400–4000 cm^−1^. The infrared spectrometer adopts transmission mode and accumulates 64 scans with a resolution of 4 cm^−1^.

### 2.5. Toxicity Studies

#### 2.5.1. Algal Cultures

Microalga cells were grown in Erlenmeyer flasks of 250 mL capacity, containing 100 mL of F/2 culture medium [23] inoculated at 10% (*v*/*v*) with exponentially growing precultures as commonly reported [24]. The microalgae grew for 18 days under continuous stirring. Cultures were maintained at 25 ± 2 °C under continuous light (80 µmol photons·m^−2^·s^−1^).

#### 2.5.2. Growth Kinetics

Cell numbers were determined using a Malassez blade under an inverted microscope (Motic microscope AE 2000, Matic microscope AE 2000, Barcelona, Spain) after formaldehyde treatment.

All concentrations were tested alongside an untreated control group in triplicate for each experiment.

#### 2.5.3. Determination of IC_50_ Value

The 50% (IC_50_) inhibitory concentrations of Ben-Ag and Ben-Ag (H_2_) against *Chlamydomonas* sp. were evaluated as previously described [25]. Sterile glass tubes containing 10 mL of culture medium were inoculated with microalgal cells in the exponential growth phase to attain an initial cell density of 10^6^ cells/mL. Synthesized nanocomposites were added to obtain different concentrations ranging from 100 to 10,000 mg/L. Unlike conventional protocols where nanoparticles are predispersed in deionized water or culture medium before exposure, in this study, the nanoparticle powder was directly resuspended in the culture medium at the time of exposure. This approach ensured immediate and direct contact between the nanoparticles and the microalgal cells throughout the experiment, better reflecting real-world environmental conditions. Tubes were incubated at 25 ± 2 °C in a rotary orbital shaker at a speed of 150 rpm, under continuous light (80 µmol photons·m^−2^·s^−1^). To prevent cell clumping, cultures were vigorously agitated once every 24 h during the exposure period. After 72 h, cell enumeration was performed as previously detailed. Subsequently, the IC_50_ values were estimated utilizing the AAT Bioquest IC_50_ calculator available at https://www.aatbio.com/tools/ic50-calculator (10 April 2023). The data represent the mean values obtained from three independent experiments.

#### 2.5.4. Microalga Cell Exposure Procedure

*Chlamydomonas* sp. cultures aged 18 days were treated with synthesized nanocomposites, Ben-Ag and Ben-Ag (H_2_), at final concentrations of 118.2 mg/L, 354.5 mg/L, and 472.7 mg/L, which correspond to 0.5 IC_50_, 1.5 IC_50_, and 2 IC_50_ of Ben-Ag. The 0.5 IC_50_ concentration represents a sublethal dose that allows for the observation of early effects or potential adaptation mechanisms. The 1.5 IC_50_ and 2 IC_50_ concentrations correspond to higher stress levels, which are used to assess responses to increased toxicity and to identify critical toxicity thresholds. In each experiment, all concentrations were tested in triplicate alongside an untreated control group.

#### 2.5.5. Photosynthetic Pigment Quantification

Photosynthetic pigments were determined spectrophotometrically. *Chlamydomonas* sp. cultures were centrifuged and the algal pellet was suspended in 100% (*w*/*v*) absolute ethanol and kept for 24 h at 4 °C in darkness. Then, a sonication step was used: 6 cycles (5 min–40 kHz/1 min rest) at 65 °C in a thermostatic sonication bath (ISO LAB Ultasonic Laborgerate GmbH, Eschau, Germany). The mixture was centrifuged at 13,000 rpm for 10 min then the supernatant was collected. Sample absorbance was recorded at 666 nm, 653 nm, and 470 nm using methods described by Wellburn and Lichtenthaler [26] and Kumar et al. [27]. The pigment content (mg/L) was determined by the following equations:

[Chlorophyll a] (mg·L^−1^) = 15.65 × A_666_ − 7.340 × A_653_ (1)

[Chlorophyll b] (mg·L^−1^) = 27.05 × A_653_ − 11.21 × A_666_ (2)

[Carotenoids] (mg·L^−1^) = (1000 × A_470_ − 2.860 × [Chlorophyll a] − 85.9× [Chlorophyll b])/245 (3)

[Total Chlorophyll] (mg·L^−1^) = [Chlorophyll a] + [Chlorophyll b] (4)

#### 2.5.6. Cytotoxicity Analysis

After 6 h, 24 h, 72 h, and 96 h of exposure to synthesized nanocomposites, microalgal cells were harvested and the MTT assay was performed [14,28,29,30,31]. Briefly, following the exposure of microalgal cells to the nanocomposites, 500 µL of the culture was added to 20 µL of MTT solution (5 mg/mL) and incubated for 4 h in the dark. Then, the suspension was centrifuged at 8000 rpm for 8 min. The acquired precipitation was mixed with 500 µL of artificial seawater (ASW) and 200 µL of DMSO. The absorbance was measured at 570 nm using a microplate reader MUTISKAN FC (Thermo Fisher Scientific, Waltham, MA, USA).

#### 2.5.7. Genotoxicity Assay and Apoptotic Potential Determination

Acridine orange staining was conducted to assess the genotoxic potential of the synthesized nanocomposites [30,31]. Briefly, 18-day-old cultures cells were treated with synthesized nanocomposites for 24 h. The microalga cells were then stained with acridine orange, Bio Basic Canada Inc., Markham, ON, Canada (7.5 mg/mL in PBS) for 5 min at room temperature. Fluorescence microscopy was performed using the FLoid^®^ Cell Imaging Station, Carlsbad, CA 92008 USA, with red and green filters set at 560 nm and 470 nm, respectively [14]. Propidium iodide (PI)-stained cells’ fluorescence (Biotium, Fremont, CA, USA) was applied in this study to assess the apoptotic potential of cells. This method allows the distinction between non-fluorescent living cells and dead fluorescent cells [32]. Briefly, after 96 h of exposure, cells were harvested using centrifugation (3500 rpm, 10 min), washed with phosphate buffer solution (0.1 M, pH 7.0), and stained with 10 μL PI for 20 min. Following incubation, the stained cells were examined under a fluorescence microscope (FLoid^®^ Cell Imaging Station) using a red filter at 560 nm.

#### 2.5.8. Protein Extraction

After dissolving 100 mg of freshly frozen microalgae samples in 1 mL of PBS (0.1 M, pH 7), the samples were subjected to 5 min of ultrasound treatment (20 kHz, at amplitudes of 90%) [33]. The homogenate was centrifuged for 20 min at 4 °C at 8000 rpm. Supernatants containing soluble proteins were collected and protein concentration was determined using the Lowry method [34], standardizing on bovine serum albumin [35].

#### 2.5.9. Estimation of Enzymatic Antioxidant Activities

Enzymatic antioxidant activities were determined to estimate the oxidative stress induced by the exposure to newly synthesized nanocomposites. The antioxidant activities of enzyme extracts and non-enzymatic antioxidants were analyzed. Glutathione peroxidase (GPX) activity was determined using the protocol reported by Flohé and Günzler [36]. Lipid peroxidation was estimated as follows: 100 mg of fresh microalgae biomass was homogenized with 1 mL Trichloroacetic acid 98% (TCA, LObachemie, Mumbai, India (0.1%), then centrifuged for 20 min at 12,000 rpm. Then, 0.5% TBA was added to the supernatant. The mixture was incubated for 30 min in a water bath at 95 °C, then fast cooled for 10 min. The content materials of MDA-TBA complexes were analyzed through measuring the absorbance at wavelengths of 532 nm and 600 nm. The MDA content material is calculated with the following formula. It is expressed in nmol/g of fresh material.

[MDA] = [A_532_ − A_600_/155,000] × 106 × Vex/g FW (5)

where Vex, FW represent volume of the extract and fresh weight, respectively, and 155,000 M^−1^ cm^−1^ corresponds to the molar extinction coefficient.

Glutathione (GSH) assay was carried out according to the protocol established by Tietze [37].

#### 2.5.10. Total Phenol Assay

Total phenol content was measured using the Folin–Ciocalteu method [38]. Briefly, 1 mL of the microalgae suspension was centrifuged at 13,000 rpm for 10 min at 4 °C. The obtained pellet was weighed, then dissolved in 1 mL of absolute ethanol (SHAMLAB, Dresden, Germany) [39]. The obtained suspension underwent ultrasonic treatment in a thermostatic sonication bath (ISO LAB Ultrasonic Laborgerate GmbH). The homogenate was centrifuged at 13,000 rpm for 10 min at 4 °C. The supernatant was recovered and mixed with 0.5 mL of Folin–Ciocalteu’s reagent (Lobachemie, Mumbai, India) then incubated, in the dark, at room temperature for 1 h. Absorbance at 750 nm was recorded. Total phenol content was expressed as mg/g of gallic acid equivalents (mg GAE/g) of fresh weight.

### 2.6. Polysaccharide Content

Polysaccharide content was determined using the phenol–sulfuric acid method, following Dubois et al. [40]. First, 1.5 mL of fresh microalgae culture was centrifuged at 12,000 rpm for 10 min and the microalgal biomass was collected. The pellet was weighed, then dissolved in 100 µL of Milli-Q water (Liqui pro, Sfax, Tunisia). The mixture was transferred into a glass hemolysis vial, and 100 μL of 5% (*w*/*v*) phenol was added. The mixture was incubated on ice for 5 min. Next, 500 µL of concentrated sulfuric acid was added, followed by vortexing. The mixture was then incubated at 100 °C in the dark for 5 min. After cooling in the dark, the optical density at 492 nm is measured. A D-glucose solution (Sigma Aldrich, Germany) was used to prepare a standard range concurrently [24,25].

### 2.7. Lipid Content

Total lipid content was determined using a chloroform (Navochim, Marseille, France)–methanol (Carlo Erba reagent, Val-de-Reuil, France)–water mixture [41]. Briefly, 50 mg of freeze-dried microalgae was dissolved in 800 µL Milli-Q water, 2 mL chloroform, and 1 mL methanol and the mixture was vigorously shaken for 2 min. Subsequently, 2 mL of Milli-Q water and 2 mL of chloroform were added, and the mixture was shaken for an additional 2 min. The suspension was then centrifuged for 10 min at 6000 rpm. The organic phase containing the lipids was collected and transferred to predried, preweighed tubes. The remaining aqueous phase and biomass residues were extracted twice more, using 2 mL of chloroform each time. To remove the solvent, the organic phases were combined and evaporated until the weight stabilized. The lipid content was determined by weighing the residue. The percentage of lipid content was calculated using the following equation:

Lipid content (%) = Weight of lipid (g)/Weight of dried microalgae biomass (g) × 100(6)

Fatty acid methyl esters (FAMEs) were prepared and analyzed using a gas chromatography–flame ionization detector (GC–FID), Shimadzu 17 A, California, USA [19].

### 2.8. FTIR Analysis

After 96 h, microalgae cells were collected by centrifugation. FTIR analysis was performed using an Agilent Technologies spectrometer Cory630FTIR (Santa Clara, CA, USA), at a resolution of 4 cm^−1^ with 10 scans in the range of 500–4000 cm^−1^.

### 2.9. Gene Expression Assessment

Fresh microalga cells were harvested by centrifugation at 6000 rpm for 10 min at 4 °C and used for total RNA extraction using an RNeasy Plant Mini Kit Isolation Kit (Qiagen, Invitrogen, Venio, Netherlands, USA) according to the manufacturer’s protocol. The resulting total RNA samples were analyzed using agarose gel electrophoresis (2%), and their purity and quantity were determined using a NanoDrop 2000 spectrometer (Thermo Fisher Scientific, Waltham, Massachusetts, USA) [14,42]. One microgram of each total RNA sample was used for cDNA synthesis using the PrimeScript™ RT Reagent Kit (Perfect Real Time) with gDNA Eraser (Perfect Real Time) (Takara, Kyoto, Japan) according to the manufacturer’s procedure.

Real-time qPCR was conducted using primer pairs summarized in Table 1.

All qPCR assays were carried out on a StepOnePlus™ PCR cycler (Applied Biosystems, Foster City, CA, USA) [46]. Amplification reactions were performed in a 10 μL reaction mix containing 5 μL of SYBR Premix Ex Taq II (2×) (Takara, Kyoto, Japan), 1 μM of each primer (Bio Basic, Canada, Inc.), and 1 μL of each appropriate cDNA sample. The used cycling conditions were 30 s at 95 °C, followed by 40 cycles of 15 s at 95 °C and 1 min at 60 °C [14,35,39]. At the end of the qPCR cycles, the amplification specificity of each primer pair was verified through a fusion step performed by heating from 60 °C to 95 °C. The gene coding for β-tubulin was used as a housekeeping gene [45]. A reaction having amplification efficiency between 95% and 105% was considered acceptable [47]. Relative mRNA expression values were determined with the 2^−ΔΔCt^ method [48].

### 2.10. Statistical Analysis

The research involved conducting three repetitions of the experimental evaluations. The GraphPadPrism software (version 8.0.1) (La Jolla, CA) was used to perform analysis of variance. We considered *p* values that were less than 0.05 as indicating significance.

The data show a high level of statistical significance between control and treatment (**** *p* < 0.0001; *** *p* < 0.001; ** *p* < 0.01; and * *p* < 0.05). A, AA, AAA, AAAA are the significance between concentrations of 0.5 IC_50_ and 1.5 IC_50_ and 2 IC_50_ (AAAA = *p* < 0.0001, AAA = *p* < 0.001, AA = *p* < 0.01, A = *p* < 0.05) measured by one-way ANOVA with Tukey’s posthoc tests.

## 3. Results and Discussion

### 3.1. Synthesis and Characterization of Nanocomposites

#### 3.1.1. Chemical Composition and Characterization

**Figure 1:**
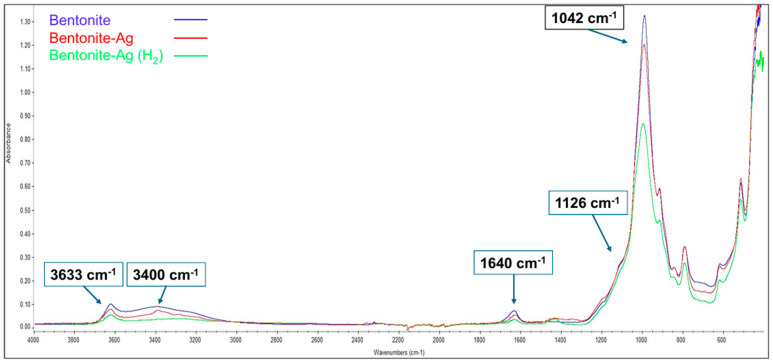
FTIR spectra of bentonite, bentonite-Ag, and bentonite-Ag (H_2_).

The silver content was estimated to be 10.33 wt% on the clay, as obtained through elemental gravimetric analysis. As a general feature, the FTIR spectra (Figure 1) of all samples showed peaks at 3633 and 3400 cm^−1^ related to the O-H stretching vibrations of the bentonite sample.

The study of Vicente-Rodríguez et al. [49] indicates that the band at 3631 cm^−1^ in the OH stretching region is attributed to hydroxyl groups bound to Al^3+^ cations present in the clay. The band at 1042 cm^−1^ is attributed stretching modes of Si-O-Si species of the clay, whereas the band at 524 cm^−1^ is related to Si-O-Al bending vibrations. The band at 1126 cm^−1^ is attributable to the out-of-plane Si-O-Si stretching mode of Si-O bonds. The band at1640 cm^−1^ is the stretching vibration of hydroxyl groups of water molecules (δ H–O–H) still physisorbed onto the clay. There was no noteworthy difference between bentonite-Ag, bentonite-Ag treated with H_2_ (Ben-Ag (H_2_)), and bentonite, suggesting that Ag^+^ ion exchange or Ag NPs did not generate new functional groups. The results obtained in this study are consistent with those available in the literature [50,51].The size of the whole support clay materials was measured by using DLS analysis (Table 2).

The size distribution of the pristine clay shows a dominant particle size around 154 nm, with a secondary peak around 803 nm (see Supplementary Materials Figure S1a). After cationic exchange with AgNO_3_ to obtain bentonite-Ag, an extensive coalescence of the small clay particles takes place and the majority of the material shows a hydrodynamic diameter that is far larger (792 nm), even after intermediate sonication (see Supplementary Materials Figure S1b). Finally, after the solid is treated under H_2_, the particles can be efficiently dispersed again and the average main size is around 161 nm, with a fully comparable size to the pristine clay support, along with a minor component of particles with a size of ca. 1030 nm (see Supplementary Materials Figure S1c). These results display that the cationic exchange with Ag^+^ salts leads to a coalescence of the fine particles due to the lower electrostatic repulsion forces caused by the extensive presence of Ag^+^ species [52]. When Ag(I) species are reduced to metallic Ag, under H_2_, the smaller particles can freely disperse again, showing an average main size of ca. 160 nm.

CHN studies showed C (0.17%), H (1.18%), N (1.22%) in Ben-Ag, C (0.24%), H (0.97%), N (0.94%) in Ben-Ag (H_2_), and C (0.23%), H (1.54%), N (1.54%), H (0%) in bentonite clay (Table 3). The nitrogen content of the exchanged materials is within instrumental error and equal to the nitrogen content of the original material.

#### 3.1.2. EDX Analysis

**Figure 2:**
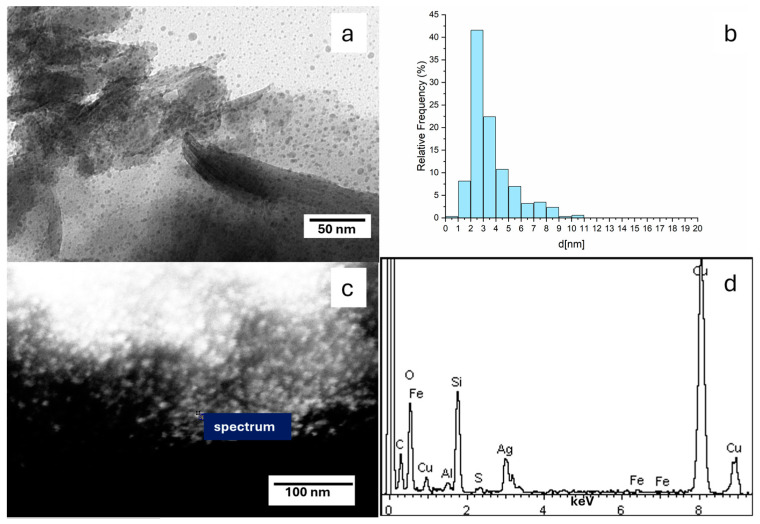
TEM and EDX characterization of bentonite-Ag sample: (**a**) TEM micrograph showing the Ag nanoparticles’ morphology and distribution, (**b**) Ag NP size distribution histogram; (**c**) STEM images showing the spot where the EDX analysis was performed; (**d**) EDX spectrum collected at the spot reported in (**c**).

We characterized the Ben-Ag sample morphologically with conventional TEM (Figure 2a) as well as with STEM (Figure 2c). In order to confirm the composition of the metal nanoparticles (NPs) observed in the micrographs, we analyzed EDX spectra of the NPs on the Ben-Ag sample (Figure 2d). The spectra confirm that the NPs are composed of Ag(0) species (Figure 2d), likely obtained by in situ redox reactions between Ag(I) species and Fe(II) sites present within the pristine natural bentonite clay.

#### 3.1.3. TEM Micrograph Study

**Figure 3:**
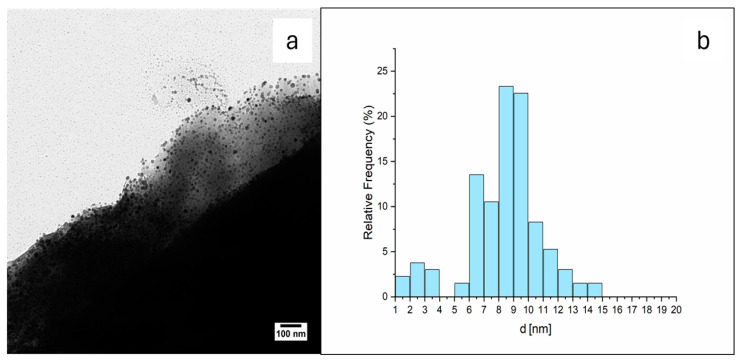
(**a**) TEM image of Ben-Ag (H_2_) sample. (**b**) Ag NP size distribution histogram. The TEM images of Ben-Ag and Ben-Ag (H_2_) samples show that bentonite is mainly composed of layered structures with slightly different interlayer spacings (Figure 2, Figure 4a,b and Figure 5).

The samples of Ben-Ag and Ben-Ag (H_2_), as said above, were morphologically characterized by TEM (Figure 2a and Figure 3a). Both samples show Ag NPs on the surface of the bentonite. The Ag NP mean size increases from 3.4 nm, with a minimum value of 1.5 to a maximum of 10.6 nm, before H_2_ treatment to 8.6 nm with a minimum value of 1.5 to a maximum of 14.8 nm. The size distribution histograms of NPs of Ben-Ag and Ben Ag (H_2_) are reported in Figure 2b and Figure 3b. It is evident that the H_2_ treatment induces the Ag-NP growth.

**Figure 5:**
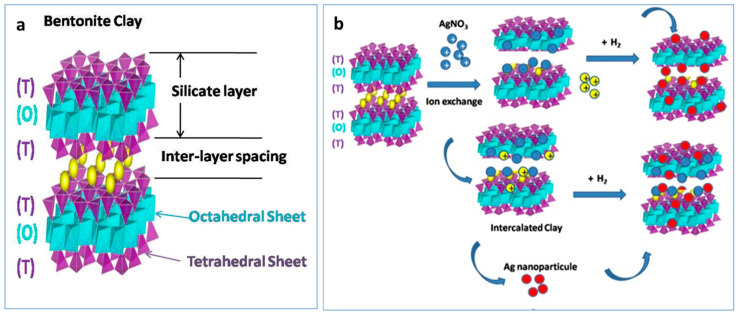
Scheme illustrating the structure of bentonite clay (**a**) and bentonite-Ag clay material formed via ion exchange method and generation of supported Ag NPs over bentonite (**b**). yellow balls is Ca^2+^, Na^+^.

Measuring the distance between the bentonite lamellae (Figure 5 and Figure S1) by TEM, mean distances of 1.6 nm (minimum distance 0.8 nm and maximum 3.0 nm) in Ben-Ag samples and 2.9 nm (minimum distance 1.7 and maximum 4.7 nm) after H_2_ treatment were obtained. This suggests that the growth of the AgNPs also influences the clay morphology.

### 3.2. Cytotoxicity Assays

#### 3.2.1. IC_50_ Value Determination

**Figure 6:**
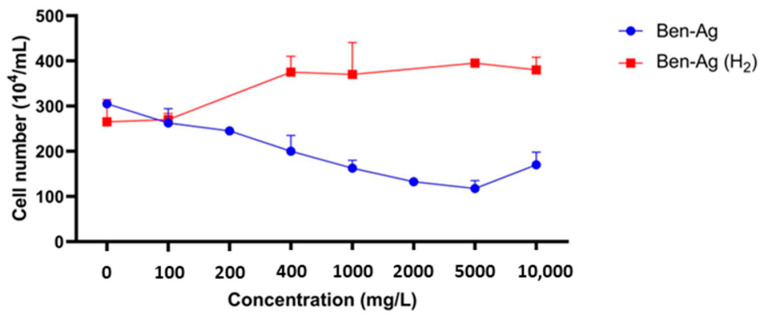
Effects of synthesized nanocomposite concentrations on the growth rate of *Chlamydomonas* sp.

The growth inhibition of *Chlamydomonas* sp., relative to the control, was assessed after 72 h of exposure of microalga cells to the synthesized nanocomposites (Figure 6).

The obtained results showed that treatment with Ben-Ag affected *Chlamydomonas* sp. growth. The IC_50_ value obtained for Ben-Ag was 236.3 ± 6.9 mg/L. Although this value may appear high and is unlikely to be attainable in environmental exposure scenarios, we must consider that Ben-Ag composites contain only 10.33% active metal (*v. supra*). Therefore, the actual IC_50_ with respect to nanostructured Ag content is ca. 24 mg/L only. Such a concentration value may be found in close proximity to target organisms in environmentally contaminated areas, where nano-Ag-containing composites were released (such as those found in Ag-containing biomedical devices or in self-decontaminating materials). The toxicity of silver nanoparticles to aquatic invertebrates and algae has been studied extensively. Pham [53] investigated the toxicity of Ag NPs to *Scenedesmus* sp. and *Thalassiosira* sp. (72 h test) and obtained IC_50_ values of 89.9 ± 9.7 and 107.2 ± 7.4 mg/L, respectively. In concordance, Ag NPs appeared to be highly toxic to the freshwater algae *Pseudokirchneriella subcapitata* and *Skeletonema costatum* with IC_50_ values of about 1.63 mg/L and 3.1 mg/L, respectively [54,55]. Ag NPs exhibit a concentration-dependent inhibition of microalgae growth [55]. Many authors speculate that the toxicity of silver nanoparticles stems from the release of elevated concentrations of silver ions upon contact with algal cells [54]. However, a clear dose–response relationship was not observed under most experimental conditions. This phenomenon can be attributed to several key properties of Ag NPs. First, Ag NPs tend to agglomerate in biological media, reducing their bioavailability and resulting in heterogeneous cellular exposure [56]. This non-uniform distribution complicates the observation of a linear dose–response relationship. Additionally, Ag NPs partially dissolve, releasing silver ions (Ag^+^), which are more toxic than the nanoparticles themselves [57]. However, the release rate of Ag^+^ depends on multiple factors, such as pH, temperature, and the composition of the biological medium, making dose-dependent effects unpredictable. Indeed, at high concentrations, Ag NP aggregation can slow Ag^+^ release, reducing toxicity instead of increasing it. Another factor to consider is the cellular uptake mechanism, which is limited by endocytosis and passive diffusion [58]. Once a saturation threshold is reached, increasing the dose does not necessarily intensify the cellular response. Moreover, Ag NPs can induce oxidative stress, triggering cellular defense mechanisms such as antioxidant production and DNA repair. At low doses, these protective responses mitigate damage, resulting in minimal observable effects, whereas at higher doses, cytotoxicity may occur in a non-linear manner [59].

In contrast, a positive effect on the growth rate for *Chlamydomonas* sp. was shown following its exposure to Ben-Ag (H_2_) (Figure 6). The lack of toxicity of Ben-Ag (H_2_) could be linked to the increased size of nanoparticles after H_2_ treatment. Several previous works have proven that nanoparticle size accounts for differences in toxicity [60,61,62]. In fact, smaller silver nanoparticles are more toxic [63]. Indeed, the toxicological effect of nanoparticles increases with decreasing particle size and increasing surface area [64]. Other studies have shown that the size of the nanoparticles is crucial for their internalization, so the toxicity will be linked to this characteristic [65,66].

In addition, the lack of toxicity observed with Ben-Ag (H_2_) may also be attributed to the phenomenon whereby, in the presence of high concentrations of nanosized silver, numerous colloids can interact and form larger aggregation complexes [67,68]. These complexes may experience significant challenges in penetrating algal cells.

#### 3.2.2. Effects of Ben-Ag on *Chlamydomonas* sp. Growth and Photosynthesis

**Figure 7:**
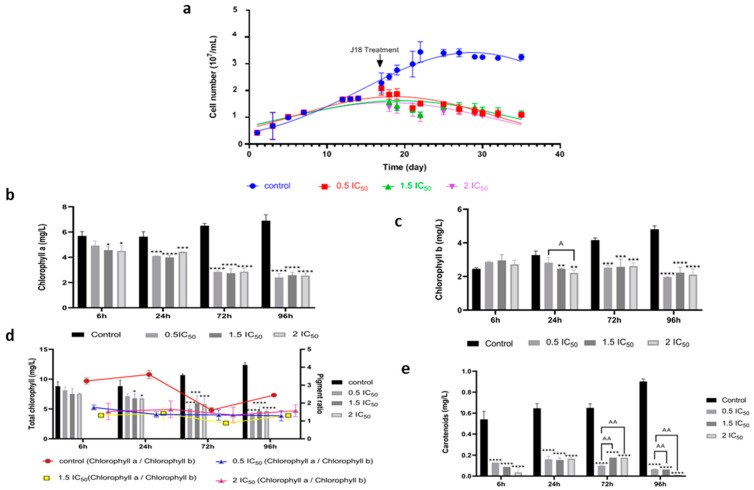
Effect of Ben-Ag on the growth and photosynthesis of *Chlamydomonas* sp. cells. (**a**) Growth, (**b**) Chlorophyll a content, (**c**) Chlorophyll b content, (**d**) Total chlorophyll content and pigment ratio, and (**e**) Carotenoid content. Statistical significance is indicated as follows: (**** *p* < 0.0001; *** *p* < 0.001; ** *p* < 0.01; and * *p* < 0.05) and (AA = *p* < 0.01; A = *p* < 0.05).

The effect of three Ben-Ag concentrations on *Chlamydomonas* sp. strain growth rate is illustrated in Figure 7a. An inhibiting effect was observed after Ben-Ag addition at all tested concentrations.

In agreement, Nazari et al. [6] reported the impact of synthesized silver-reduced graphene oxide nanocomposites on the growth rate of *C. vulgaris*. They show that biomass was significantly reduced after cells’ exposure to nanocomposites at concentrations of 2, 4, and 6 mg/L compared to the untreated control [6]. Furthermore, Dedman et al. [69] demonstrate that Ag nanoparticles and ionic silver reduce populations of the cyanobacterium *Prochlorococcus* by more than 90% at concentrations ≥10 μg/L. It is worth noting that Ag nanoparticles exert their toxicity by releasing toxic silver ions into the medium, while other forms (PEG- or PVP-coated, different sizes) of Ag nanoparticles have negligible toxicity [70].

After exposing *Chlamydomonas* sp. cells to Ben-Ag for 6 h, 24 h, 72 h, and 96 h, a significant decrease in chlorophyll a, chlorophyll b, and total chlorophylls was observed at all concentrations tested from 24 h of exposure, compared to the samples treated with the control (Figure 7b–d). However, a decrease in carotenoid content was observed starting from 6 h (Figure 7e). Previous reports confirm that the decrease in chlorophyll a content increased with increasing concentrations of Ag nanoparticles and Ag^+^ ions. In fact, Khoshnamvand et al. [71] reported the growth inhibition of *Chlorella vulgaris* in the presence of Ag nanoparticles and Ag^+^ ions. In addition, Książyk et al. [54] reported that Ag nanoparticles, at concentrations of 5, 10, 15, 20, and 25 mg/L, cause a 100% drop in chlorophyll a and chlorophyll b, as well as total chlorophylls. Chlorophyll has been widely used as a toxicity indicator, since it is an important photosynthetic pigment essential for algal cell function. When the particles enter the cell, the reachability of light is reduced, thus reducing the process of energy transfer [72]. Cell growth and chlorophyll levels are impaired as a result of the effect of oxidative stress [72]. Similarly, in the plant *Lemna minor*, chlorophylls a and b contents were reduced as a function of the duration of exposure to iron oxide nanoparticles and also concentration [73].

Calculated chlorophyll a:chlorophyll b ratios (Figure 7d) were stable in microalgae cells treated with 0.5 IC_50_ and 1.5 IC_50_ Ben-Ag exposure. The application of a Ben-Ag concentration equal to 2 IC_50_ caused a greater reduction in chlorophyll b compared to chlorophyll a after 6 h, 24 h, and 96 h, which explains why the chlorophyll a:chlorophyll b ratio tended to increase. Decreased chlorophyll level is one of the most important indicators of oxidative stress [27,39,74]. Disruption of photosynthetic pigments may be primarily due to disturbances in the electron transport chain and displacement of Mg^2+^ ions associated with the tetrapyrrole ring of the chlorophyll molecule. Indeed, tetrapyrrole-dependent and -independent mechanisms for reducing ROS generation and accumulation are examples of induced oxidative stress responses. As a result, tetrapyrroles can contribute to oxidative stress but also play a crucial role in the detoxification of reactive oxygen species (ROS), which protects cells during the oxidative stress response [75].

#### 3.2.3. Effects of Ben-Ag on Cell Viability

**Figure 8:**
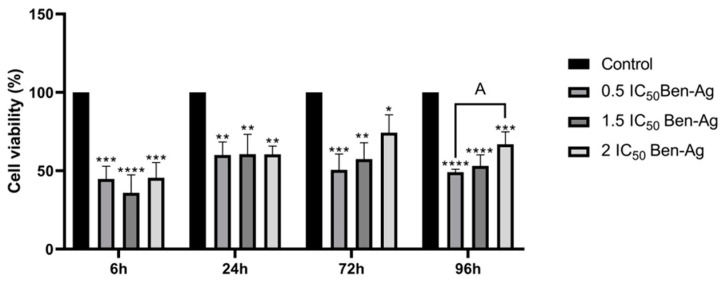
Effects of Ben-Ag on cell viability. Statistical significance is indicated as follows: (**** *p* < 0.0001; *** *p* < 0.001; ** *p* < 0.01; and * *p* < 0.05). A is the significance between concentrations of 0.5 IC_50_ and 1.5 IC_50_ and 2 IC_50_ (A = *p* < 0.05).

The cytotoxicity induced by the Ben-Ag in the microalgal cells was evaluated, after 6 h, 24 h, 72 h, and 96 h, using the MTT test. Figure 8 shows a decrease in cell viability after 6 h and 24 h. There was a slight rise in cell viability as a function of exposure concentration after 72 h and 96 h. A slight increase was seen at higher concentrations (2 IC_50_) after 72 h and 96 h, but the presence of 0.5 IC_50_ was associated with a decrease in cell viability.

The fact that silver nanoparticles, like other kinds of nanoparticles, are insensitive to potential forces acting on them in their surroundings may account for the observed lack of toxicity. Thus, Brownian movements are the only explanation for the movements of these nanomaterials. It is possible that multiple nanoparticles will come into contact and aggregate during these movements, altering their toxicological and physical characteristics. Furthermore, it is common for silver nanoparticles to dissolve in solutions, indicating their release into the medium of silver ions whose toxicity is proven at low concentrationsinbacterial communities [76]. The effect of silver nanoparticle concentration on aggregate size distribution was demonstrated by Millour et al. [77]. It is found that the particle concentration is a limiting factor for aggregation because, at mg L^−1^ levels, over 80% of aggregates have a size larger than 200 nm, but at µg·L^−1^ levels, over 60% of aggregates have a size smaller than 200 nm. Furthermore, aggregation may have an impact on nanomaterials’ environmental fate and toxicity mechanisms. Different forms of silver, such as dissolved silver, AgNPs, or colloidal silver, could be produced by aggregation [68]. Large colloidal aggregates cannot directly pass through the cell membrane, but small aggregates can interact with the cellular surface and be internalized [78]. Additionally, small colloidal aggregates have the ability to stay in suspension for extended periods of time, increasing the possibility of interactions with other colloids (such as detritus or living cells) or even transport far from their discharge point in the environment. In contrast, large colloidal aggregates can settle quickly [79].

According to Figure 8, the Ben-Ag cytotoxicity changes in a time-dependent manner. Indeed, there is a decrease in cell viability after 6 h to less than 50% for all concentrations and there is an increase in cell viability after 72 h and 96 h to higher than 50%. Different silver nanoparticle aggregation states were developed, and the aggregation-dependent toxicity was demonstrated by Bélteky et al. [80]. The authors disclosed that cell viability increased and nanoparticle toxicity decreased with growing aggregation grade. These changes in AgNP cytotoxicity were caused by particle aggregation in a time-dependent manner. In fact, as aggregation time increased, cell viability increased as well.

In this context, Nazari et al. [81] showed that cell viability of the green microalga *Chlorella vulgaris* was reduced to 57.82% after 24 h of exposure to reduced graphene oxide silver (Ag-GO) nanocomposites at a concentration of 30 mg/L. Ag nanoparticles was also found to be more cytotoxic than asbestos regarding their ability to inhibit macrophage viability [82].

### 3.3. Effects of Ben-Ag on Enzymatic Antioxidant Activities and Non-Enzymatic Antioxidants

**Figure 9:**
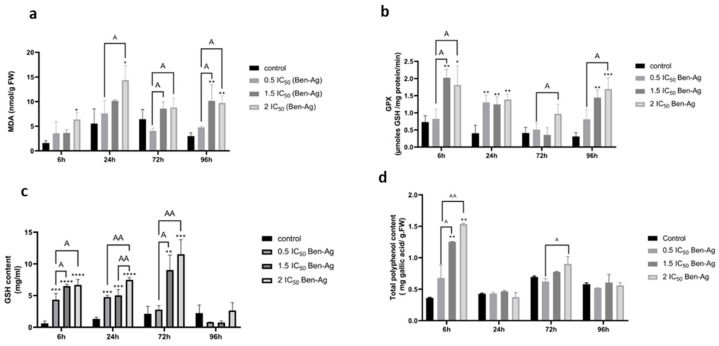
Antioxidant Enzyme Activities and Non-Enzymatic Antioxidant Levels in Algal Cells Treated with Ben-Ag. (**a**) MDA content, (**b**) GPX content, (**c**) GSH content, and (**d**) Total polyphenol content. Statistical significance is indicated as follows:(**** *p* < 0.0001; *** *p* < 0.001; ** *p* < 0.01; and * *p* < 0.05) (AA = *p* < 0.01; A = *p* < 0.05).

ROS formation is a biochemical change that is thought to be caused by exposure of a living organism to harmful stress [83]. The intracellular producers of ROS in plant cells are chloroplasts and mitochondria [84]. The antioxidant defense system is critical to counteract the harmful effects of ROS. Therefore, changes in activity of some antioxidant enzymes such as CAT, APX, GPX, and SOD can be used to assess cellular damage [14]. The results of the enzymatic activity tests revealed that microalgal cells treated with Ben-Ag showed a significant increase in MDA activity after 24 h and a significant increase in GPX activity after 6 h at all tested concentrations compared to the negative control (Figure 9a,b).

In this context, an increase in MDA activity was also observed in *Scenedesmus obliquus* and *Chlamydomonas reinhardtii* exposed to graphene oxide nanocomposites reduced by metal nanomaterials [85]. Moreover, excess ROS have been shown to induce multiple kinds of damage in algal cells, resulting in dysfunction of many organelles and fundamental processes. Assuming that the MDA content is proportional to the rate of free radicals, we can conclude that Ben-Ag stimulates the free-radical-generating capacity of *Chlamydomonas* sp.

In parallel with enzymatic antioxidant defense systems, microalgae possess non-enzymatic protection systems against ROS, including production of phenolic compounds and low-molecular-weight compounds such as glutathione (GSH). Upon exposure of microalgal cells to Ben-Ag at concentrations of 1.5 IC_50_ and 2 IC_50_, GSH levels significantly increased after 6 h, 24 h, and 72 h compared to untreated controls (Figure 9c). A concentration-dependent decrease in GSH accumulation was evident when exposed to Ben-Ag (Figure 9a).

Phenolic compound stimulation was also observed after 6 h of Ben-Ag treatment (Figure 9d). However, at 72 h and 96 h there was no significant difference between Ben-Ag-treated cells and negative control. Several studies reported that oxidative stress triggered an induction of the detoxification system, with an increase in GSH, GST, GPX, and MDA levels under metal oxide (Fe_2_O_3_)NP exposure in the terrestrial snail *Helix aspersa* [86]. Activation of a plant’s secondary metabolism is the main defense mechanism and is crucial for the synthesis of phenolic compounds. Phenolic compounds play an important role in ROS detoxification and also act as electron donors in the detoxification mechanisms of organelle structures [87].

### 3.4. FTIR Spectra of Chlamydomonas sp. After Exposure to Ben-Ag

**Figure 10:**
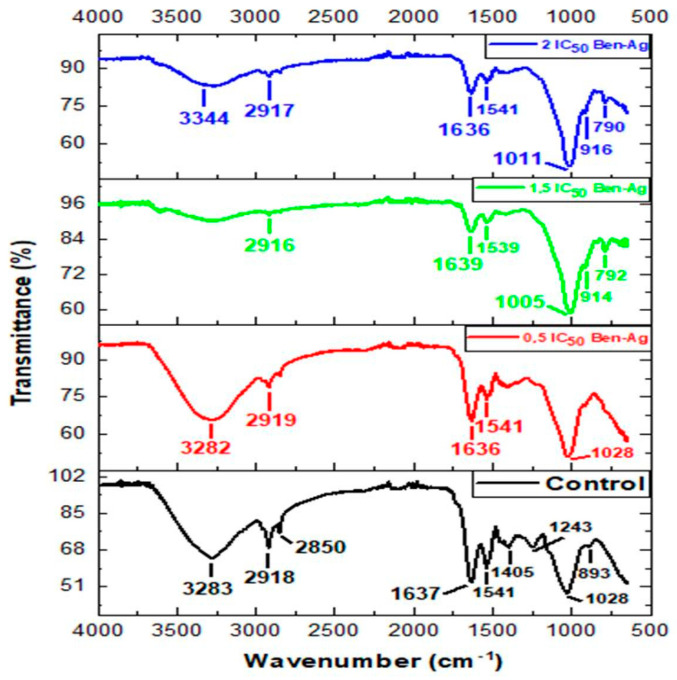
FTIR spectra of *Chlamydomonas* sp. after exposure to Ben-Ag.

FTIR analysis was performed to determine the functional groups of cell wall biomolecules after interacting with Ben-Ag (Figure 10).

The band at around 3282 cm^−1^ refers to the symmetric O-H and N-H stretching of hydroxyl and amide from water and proteins [88]. We found the presence of asymmetric CH_2_ stretching of methyl groups at ca. 2918 cm^−1^ belonging to the long methylene chains of the lipid fraction [89,90]. The peaks at 1639 and 1636 cm^−1^ are symmetric C=O stretching modes of protein amide I, whereas symmetric deformations of NH bending and C-N stretching modes of protein amide II are found between 1541 and 1539 cm^−1^ [85,86]. The peak at 1405 cm^−1^ is attributed to the C-N stretching mode of amide II (protein). The peak at 1243 cm^−1^ was associated with asymmetric P=O stretching of phosphodiesters of nucleic acids and phospholipids [89]. Furthermore, the region between 1028 and 1011 cm^−1^ may be related to the presence of carbohydrate species with CO and polysaccharide C-O–C stretching modes [91,92]. In contrast to control cells, the ones treated with Ben-Ag show a new band at 3344 cm^−1^ probably due to the presence of Ag-OH species. Fazelian et al. [93] suggested that exposure of *Nannochloropsis oculata* to AgNPs peaked at 3412 cm^−1^ representing Ag-O-H groups. The peak at 1028 cm^−1^ is related to the CO stretching mode of alcohol groups and was shifted to form bands at 1011 cm^−1^ and 1005 cm^−1^ in Ben-Ag-treated cells, thus suggesting a modification of bands due to C-O, C-O-C stretching modes of carboxyl and hydroxyl groups. In Figure 10, Ben-Ag-treated cells show a decrease in the intensity of the O-H band and a decrease in the N-H band. This indicates the interaction of the ion with the NH group via electron ion pairs of the nitrogen atom, as suggested by Ferreira et al. [94]. The decrease in relative intensity of the peaks between 1639 and 1405 cm^−1^ indicates that amides I and II are involved in Ag nanoparticle adsorption. This may be due to the interaction of amide groups with cations characterized by electron ion pairs over oxygen atoms and nitrogen atoms [94]. There was a change in the infrared band of the lipid region (3000–2800 cm^−1^) after treatment with Ben-Ag for 96 h. It can be attributed to changes and modifications of lipid composition. Similar changes in FTIR corresponding to protein and lipid were also found in *Arthrospira platensis* under Ag nanoparticle stress [95]. There are also some peak shifts in the spectral region from 1500 to 700 cm^−1^, and a peak at 1243 cm^−1^ corresponding to the asymmetric stretching of P=O in nucleic acids was observed in the FTIR spectra of the untreated control, but it was absent in Ben-Ag-treated cells’ spectra.

### 3.5. Genotoxic and Apoptotic Potential of Ben-Ag Against Chlamydomonas sp.

**Figure 11:**
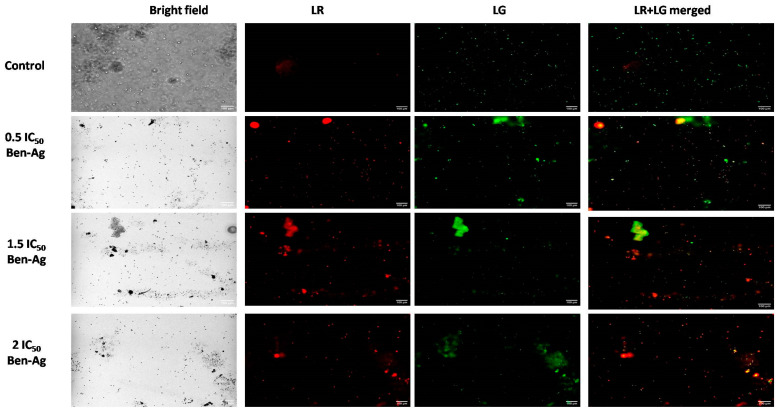
Genotoxic potential of Ben-Ag against *Chlamydomonas* sp. by acridine orange staining. Fluorescence images under green filter (LG), under red light (LR), and under two fluorescent lights (LR + LG) merged.

In this study, the acridine orange (AO) staining assay was used to evaluate the genotoxic potential of Ben-Ag against *Chlamydomonas* sp. Figure 11 shows the result of the fluorescence image obtained with AO.

Under a fluorescent microscope, cells can be divided into cells with green nuclei, corresponding to cells with intact DNA, and cells with orange or red nuclei, corresponding to damaged cells with fragmented DNA. All nuclei in control samples (appearing as green fluorescence) showed regular globular structures and chromatin organization. Nuclei of microalgal cells treated with Ben-Ag were characterized by typical morphological changes, morphological shrinkage, and changes in nucleolus position that were clearly visible in the microscopic observations (Figure 11). Over time, multiple nuclei containing fragmented DNA were visualized, appearing as red fluorescent spots. In advanced stages, the decay of apoptosis leads to complete nuclear DNA fragmentation (orange-tored-stained nuclei). The cells treated with silver nanoparticles showed green and red nuclei with nuclear condensation and cell shrinkage when stained with acridine orange/ethidium bromide which indicates that a human non-small-cell lung cancer cell line (A549) can undergo apoptosis when exposed to Ag nanoparticles at an IC_50_ concentration of 15 µg/mL [96].

**Figure 12:**
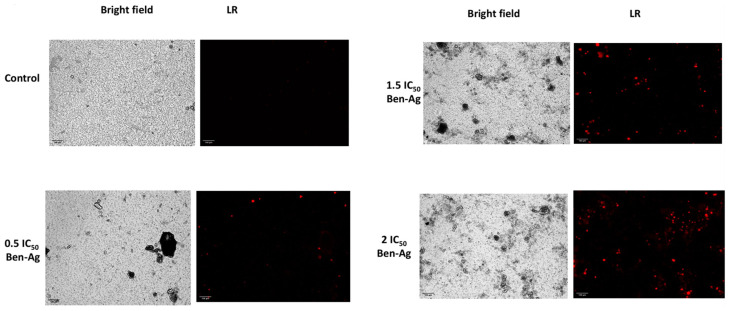
Fluorescence images of apoptotic potential of Ben-Ag against *Chlamydomonas* sp. by propidium iodide (PI) staining. Fluorescence images under red light (LR).

The *Chlamydomonas* sp. cells were exposed to varying concentrations of Ben-Ag in order to determine the underlying mechanism of cells that have lost their membrane integrity (a characteristic phenomenon of necrosis). Following treatment, the cells were stained with propidium iodide (PI). PI staining results demonstrated that Ben-Ag caused nuclear fragmentation of the *Chlamydomonas* sp. cells, a sign of apoptosis (Figure 12).

A previous study on renal epithelial cells that used nano-COM and nano-COD crystals showed that the nano-COM crystals induced higher cell death than the nano-COD crystals [97].

### 3.6. Effect of Ben-Ag Exposure on Protein, Polysaccharide, and Lipid Contents

**Figure 13:**
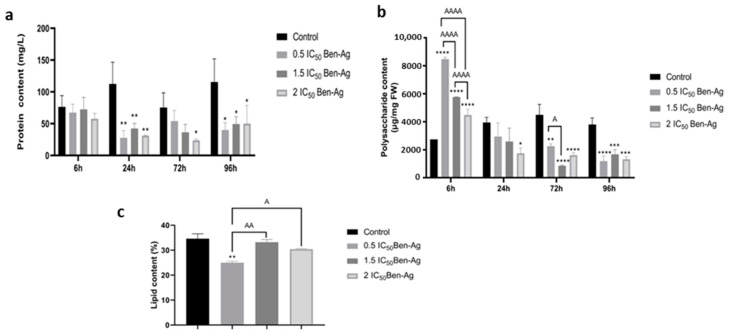
Polysaccharide, protein, and lipid contents after exposure to Ben-Ag. (**a**) Protein content, (**b**) Polysaccharide content, and (**c**) Lipid content. Statistical significance is indicated as follows: (**** *p* < 0.0001; *** *p* < 0.001;** *p* < 0.01; and * *p* < 0.05) and (AAAA = *p* < 0.0001; AA = *p* < 0.01; A = *p* < 0.05).

The effect of three Ben-Ag concentrations on protein content is illustrated in Figure 13a. The results revealed that algal cells treated with Ben-Ag showed a significant decrease in the protein content after 24 h, 72 h, and 96 h at all tested concentrations compared to the negative control. In concordance, all the tested concentrations of Ben-Ag caused significant (*p* < 0.05) loss in the protein content of *Chlamydomonas* sp. from 24 h to 96 h (Figure 13a). In concordance, Liang et al. [95] showed that the treatment of *A. platensis* with Ag nanoparticles caused a dose- and time-dependent reduction in protein content.

The exposure to Ben-Ag caused a significant increase in carbohydrates of *Chlamydomonas* sp. for the concentration of 0.5 IC_50_ at 6 h. Meanwhile, a significant decrease was observed in all the tested concentrations of Ben-Ag from 24 h to 96 h (Figure 13b). Maximum reduction in carbohydrate content was observed at 96 h. However, a study on *Skeletonema costatum* that used Ag nanoparticles at concentrations of 0.05 to 50 µg/mL showed that there was little effect on the amount of carbohydrates after 24 h [98].

The exposure to Ben-Ag caused a significant decrease in lipid content of *Chlamydomonas* sp. for the concentration of 0.5 IC_50_ at 96 h. Meanwhile, a decrease was observed in all the tested concentrations of Ben-Ag for the concentrations of 1.5 IC_50_ and 2 IC_50_ (Figure 13c). Maximum reduction in lipid content was observed at 96 h. This change in lipid content validated the latter result of FTIR and the change in the infrared band of the lipid region (3000–2800 cm^−1^) after treatment with Ben-Ag for 96 h.

Changes in fatty acid composition and synthesis are brought about in microalgae by environmental factors that induce oxidative stress [93]. Under a variety of environmental stresses, including a lack of darkness and even chemical treatment with substances, microalgae have been shown to increase their fatty acid contents [18,19].The lipid profile of *Chlamydomonas* sp. is shown in Table 4 both before and after treatment with Ben-Ag.

The lipid profile had somewhat similar changes (such as an increase or decrease in the amount of a certain fatty acid). Both short- and long-chain saturated fatty acids (C14:0–C20:0) are part of the lipid profile. When compared to the control, the Ben-Ag treatment increased the production of fatty acids such as heptadecanoic, linoleic, and linolenic acids and decreased the production of stearic and oleic acids. In comparison to the controls (46.98%), the sum of the saturated fatty acids (SFAs) increased in *Chlamydomonas* sp. following Ben-Ag exposure at 0.5 IC_50_, 1.5 IC_50_, and 2 IC_50_ (49.87%, 48.9%, and 47.15%, respectively). There was a decrease in the contents of unsaturated fatty acids (UFAs) from 55.08% to 49.86%, 51.1%, and 52.85%, respectively. The highest contents were found in the 1.5 IC_50_ and 2 IC_50_ Ben-Ag-treated algae, respectively, with palmitic acid (C16:0) at 26.77% and linoleic acid (C18:3) at 16.13%. In comparison to UFAs, MUFAs, and polyunsaturated fatty acids (PUFAs), the highest contents of PSFAs (12.46%, 9.65%, and 18.23% in *Chlamydomonas* sp.) following Ben-Ag treatment indicate that the generated lipids are resistant to auto-oxidation (peroxidation). The lipid profile analyses verified previous reports that the introduction of Ag nanoparticles and stresses modify the metabolism of numerous algal species towards the production of hydrocarbons, such as lipids [93,99].

### 3.7. Responses of Genes Linked to Oxidative Stress, Photosynthesis, Astaxanthin, and Carbohydrate Biosynthesis Pathways

To understand the transcriptional responses of microalgae exposed to Ben-Ag, the expression levels of three genes related to photosynthesis were investigated.

**Figure 14:**
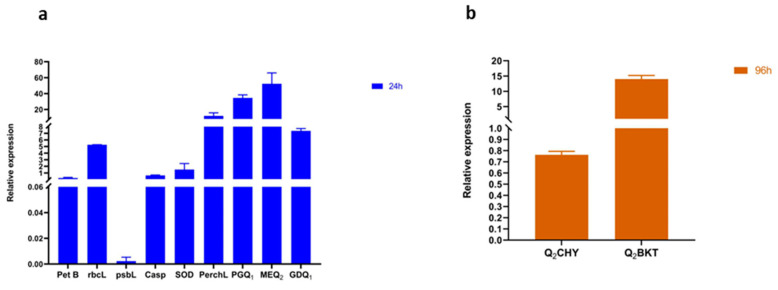
Relative expression of genes linked with photosynthesis pathway, oxidative stress, carbohydrate biosynthesis pathway, (**a**) and astaxanthin biosynthesis pathway (**b**).

The *petB* gene encoding cytochrome b (N-terminal)/b6 showed a down-regulation in microalgae cells following Ben-Ag exposure. The decreased expression of the *petB* genesuggests that Ben-Ag exposure had detrimental effects on the Cyt b6/f complex. Tikhonov [100] reported that the rate-limiting phase of the photosynthetic electron transport chain was caused by the Cyt b6/f-complex. The *rbcL* gene encoding ribulose bisphosphate carboxylase large chain (Rubisco) was up-regulated by 5-fold after 24 h of exposure (Figure 14a). According to Shayan et al. [101], Rubisco plays a key role in defense and photosynthetic regulation under moderate thermal stress [101]. The relative expression levels of the *psbL* gene, encoding P700 chlorophyll a apoproteins of the Photosystem I complex, were analyzed. Previously, many down-regulated genes were associated with light-harvesting complexes, photosynthetic electron transport systems, and transcription factors, which typically increase after 12 h of thermal stress [102]. In addition, Alafari and Abd-Elgawad [103] reported that the down-regulation of multiple photosynthetic genes, including *psbL*, *psbH*, *psbK*, *psbE*, *psbK*, *psbH*, *RCA2*, *psbI*, *psaB*, *psbD*, *psbK*, and *psaC*, and the up-regulation of *rbcl* gene may be related to *T. propinqua* seedlings’ ability to withstand heat stress. These changes are linked to processes like electron transport, the Calvin cycle, photorespiration, photosynthesis, carbon fixation, and the reductive pentose-phosphate cycle, all of which are associated with oxidative stress and the regulation of redox process. This is consistent with the fundamental function of controlled proteins and metabolites, which include the nucleus, thylakoid membrane, and chloroplast, which are connected to photosynthetic pathways.

The expression of genes related to apoptosis was also assessed in order to identify the underlying mechanisms of Ben-Ag cytotoxicity on *Chlamydomonas* sp. cells. When comparing the treated group to the control group, down-regulation of the apoptosis-related gene *casp* was recorded after 24 h of Ben-Ag exposure. Similarly, Guan et al. [104] found that TiO_2_ nanoparticle exposure reduced caspase gene expression in *Tegillarca granosa*, compromising hemocyte removal and potentially reducing phagocytic activity. Furthermore, Kulasza and Skuza [105] reported that caspase and GST proteins were down-regulated in the mussel *Mytilus galloprovincialis* after two weeks of exposure to 10 µg L^−1^ Ag nanoparticles. Previous studies identified caspase-3, caspase-6, and bcl-2 as key proapoptotic genes [106,107].

Research has shown that sublethal ROS levels act as a signal in multicellular animals and plants, triggering defense pathways. Indeed, a positive correlation has been observed between cell death and ROS accumulation. Enzymes like GPX, GST, and SOD play key roles in antioxidant defense [108]. We focused on the expression of genes involved in ROS detoxification. The superoxide dismutase (*SOD*) and peroxidase (*perchL*) genes showed 2- and 16-fold increases, respectively, after 24 h of exposure (Figure 14a). In concordance, previous studies demonstrated the overexpression of genes encoding superoxide dismutase (SOD) and peroxidase in response to exposure to Ag nanoparticles in *Arabidopsis* and cyanoacrylate resin nanoparticles (iBCA-NPs) in *Chlamydomonas reinhardtii* [108,109].

Gene expression analysis was conducted to monitor pathways involved in carbohydrate biosynthesis and determine which carbohydrates were mobilized. The phosphoglucomutase (PG) coding gene was used to track glucose biosynthesis, while the UDP-glucuronate decarboxylase (GD) coding gene was associated with xylose biosynthesis. The GDP-mannose 3,5-epimerase (ME) coding gene was examined for its role in galactose biosynthesis. Regarding the GD coding gene linked to xylose synthesis, Ben-Ag exposure resulted in a 7-fold overexpression after 24 h (Figure 14a). Likewise, overexpression of both tested genes implicated in the biosynthesis pathway of galactose and glucose after Ben-Ag exposure was observed. In fact, the ME coding gene, linked to the galactose biosynthesis pathway, showed 50-fold overexpression after 24 h. However, the expression levels of genes involved in glucose biosynthesis increased about 30-fold after 24 h. Déniel et al. [43] revealed variations in the expression of genes associated with carbohydrates under polystyrene nanoparticles in *Chlamydomonas reinhardtii*. These variations included overexpression of the genes coding for UDP-glucoronate decarboxylase, GDP-mannose 3,5-epimerase (*MEQ2*), and phosphoglucomutase (*PGQ1*), which are implicated in the biosynthesis of glucose, xylose, and galactose, respectively.

To comprehend the diverse transcriptional reactions of algae exposed to Ben-Ag, the expressions of two genes, *Q2CHY* and *Q2BKT*, associated with the astaxanthin biosynthesis pathway were evaluated after 96 h. The *Q2CHY* gene showed a 30% decrease in expression after 96 h. Meanwhile, the *Q2BKT* gene showed up-regulation followed by a 15-fold increase after 96 h (Figure 14b). Previously, Déniel et al. [43] revealed variations in the expression of genes associated with the astaxanthin biosynthesis pathway after polystyrene nanoparticle exposure in *Chlamydomonas reinhardtii*. These variations included under/overexpression of the genes *Q2CHY* and *Q2BKT*, which are implicated in the conversion of β-carotene into astaxanthin and catalyze a limited number of steps for astaxanthin synthesis, respectively.

## 4. Conclusions

The toxicities of Ben-Ag and Ben-Ag (H_2_) were evaluated against *Chlamydomonas* sp. as a model microalga. Ben-Ag materials were significantly dose-dependently toxic to *Chlamydomonas* sp. According to the obtained results, Ben-Ag affected the growth of microalgal cells. In addition, it caused an increase in MDA and GPX activities as well as phenolic compound contents. Ben-Ag induces cell morphology changes and decreases in photosynthetic pigment contents. Interestingly, an increase in lipid contents was recorded, associated with modification in fatty acid composition. These data help to fill knowledge gaps in the mechanisms of toxicity of nanostructured materials. At the molecular level, Ben-Ag appears to induce overexpression and under expression of genes encoding enzymes involved in polysaccharide biosynthetic processes, apoptosis, oxidative stress, and the astaxanthin biosynthetic pathway. Overall, this work provides a compelling argument for the ecotoxicological assessment of nanocomposites using microalgae, helping to understand their potential effects on both individual organisms and ecosystems as a whole. This is essential for preventing adverse impacts on aquatic ecosystems.

## Figures and Tables

**Figure 4:**
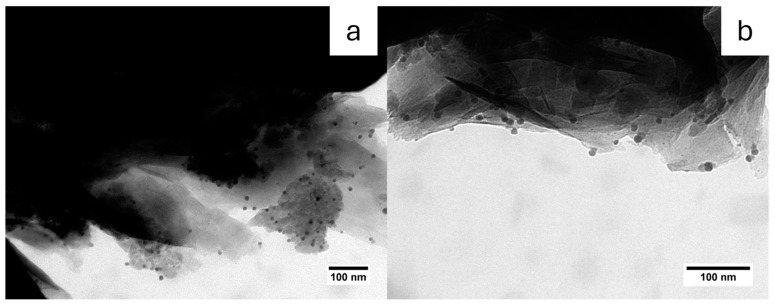
TEM image for Ben-Ag (H_2_) sample where the bentonite lamellae are visible. Different sports in (**a**,**b**).

**Table 1:** Primers used in qPCR analysis.

Metabolic Pathways	Target Genes	Primer Sequences	References
Oxidative stress	Caspase (*Casp*)	F: 5′-GCAAGAAGGCTGTCCTCATC-3′ R: 5′-GCTCTTGTCCGTGTCAATCA-3′	[43,44]
	Superoxide dismutase (*SOD*)	F: 5′-ATGAACATCCACCACACCAA-3′ R: 5′-CCTTCCAGAAGAAGCTGTGG-3′	
	Peroxidase (*PerchL*)	F: 5′-CAGCTTTCCTGCAGACCTTT-3′ R: 5′-TCCACAAACTCCTCCTCCAC-3′	
Photosynthesis	Cytochrome b(N-terminal)/b6 (*petB*)	F: 5′-GGATTGCATCAGGAACACCT-3′ R: 5′-AGGTGGTTTCAAACGTCCAG-3′	[44]
	Ribulose bisphosphate carboxylase, large chain (*rbcL*)	F: 5′-AGCTTCAGCAACGAAAAGGA-3′ R: 5′-ATTCGTAGGTCCTCCACACG-3′	
	P700 chlorophyll a apoproteins of the Photosystem I complex (*psbL*)	F: 5′-ATGGCTAGACCAAATCCAAA-3′ R: 5′-TAGAGAAAAGAACAGCTAATACGAAAA-3′	
Astaxanthin biosynthesis	β-carotene hydrolase (*Q2 CHY*)	F: 5′-GAGCTCAACGACATCTACGC -3′ R: 5′-TTGGTGTGGTGGATCTGATG -3′	[43]
	β-carotene ketolase (*Q2 BKT*)	F: 5′-TACCACTTCGACCTGCACTG-3′ R: 5′-GAGGCGGAGGAAGCTGAC-3′	
Carbohydrate biosynthesis	GDP mannose 3-5 epimerase galactose (*ME Q2*)	F: 5′-GTCCTTCGACGACAAGAAGC-3′ R: 5′-TGCTGTGGCTGTACTTGGTT-3′	[43]
	UDP-glucoronate decarboxylase xylose (*GDQ1*)	F: 5′-GTGACTACCTGGTGGCTCGT-3′ R: 5′-GATTTGGTCCACCTCCAAGA-3′	
Housekeeping gene	β-tubulin	F: 5′-TGTACGACATCTGCTTCCGC-3′ R: 5′-AGCCGACCATGAAGAAGTGC-3′	[45]

**Table 2:** Size of clay and Ag-modified Bentonite as measured by DLS (main size average and polydispersity index (PDI)).

Nanomaterials	Main Size Average (nm)	Standard Deviation (nm)	PDI
Ben	154.1	63.9	0.571
Ben-Ag	791.8	172.5	0.467
Ben-Ag (H_2_)	160.9	45.65	0.695

**Table 3:** CHN analysis.

	Bentonite	Ben-Ag	Ben-Ag (H_2_)
C (%)	0.23	0.17	0.24
H (%)	1.54	1.18	0.97
N (%)	0	1.22	0.94

**Table 4:** Fatty acid compositions.

	Culture Condition			
	Control (%)	0.5 IC_50_ Ben-Ag (%)	1.5 IC_50_ Ben-Ag (%)	2 IC_50_ Ben-Ag (%)
Myristic acid (C14:0)	4.07	2.70	2.51	2.71
Palmitic acid (C16:0)	24.10	19.65	26.77	13.70
Hepthadecanoic acid (C17:0)	10.94	16.41	8.85	26.01
Stearic acid (C18:0)	5.81	11.11	10.77	4.73
SFAs	44.92	49.87	48.9	47.15
Palmitoleic acid (C16:1)	28.11	26.49	14.55	23.42
Hepthadecenoic acid (C17:1)	2.06	2.36	1.14	3.25
Oleic acid (C18:1)	12.01	7.10	24.69	6.02
Gadoleic acid (C20:1)	3.58	1.45	1.07	1.93
MUFAs	45.76	37.4	41.45	34.62
Linoleic acid (C18:2)	3.86	2.25	5.37	2.10
Linolenic acid (C18:3)	5.46	10.51	4.28	16.13
PUFAs	9.32	12.46	9.65	18.23
UFAs	55.08	49.86	51.1	52.85

## Data Availability

The data presented in this study are available on request from the corresponding author due to the large volume of data and non-standardized format requiring additional processing before sharing.

## Supplementary Materials

The following supporting information can be downloaded at: https://www.mdpi.com/article/10.3390/nano15080629/s1. Figure S1a. DLS analysis for the sample Bentonite. Figure S1b. DLS analysis for the sample Bentonite-Ag. Figure S1c. DLS analysis for the sample Bentonite-Ag (H_2_).
