# Supplementary material for: Toxicological Effects of Silver-Modified Bentonite Nanocomposites on Microalgae: Impact on Cell Growth, Antioxidant Enzymes, and Gene Expression
Source: Nanomaterials (Basel). 2025 Apr 20;15(8):629. doi: 10.3390/nano15080629 (PMC12029818; doi:10.3390/nano15080629)
Supplement: Supplementary file 1 [file nanomaterials-15-00629-s001.zip › nanomaterials-3530688-supplementary.pdf]

nanomaterials

Supplementary Materials

Toxicological Effects of Silver-Modified Bentonite Nanocomposites on Microalgae: Impact on Cell Growth, Antioxidant Enzymes, and Gene Expression

Oumayma Ghariani [1,2], Jihen Elleuch [1], Anna Maria Ferretti [3], Stefano Econdi [2], Chiara Bisio [4], Philippe Michaud [5,*], Imen Fendri [6], Matteo Guidotti [2] and Slim Abdelkafi [1]

1 Enzymatic Engineering and Microbiology Laboratory, Algae Biotechnology Unit, National Engineering School of Sfax, University of Sfax, Sfax 3038, Tunisia; oumayma.ghariani@enis.tn (O.G.); jihen.elleuch@enis.tn (J.E.); slim.abdelkafi@enis.tn (S.A.)
2 CNR-SCITEC Istituto di Scienze e Tecnologie Chimiche "G. Natta", Via C. Golgi 19, 20133 Milano, Italy; stefano.econdi@scitec.cnr.it (S.E.); matteo.guidotti@scitec.cnr.it (M.G.)
3 CNR-SCITEC Istituto di Scienze e Tecnologie Chimiche "G. Natta", Via G. Fantoli 16/15, 20138 Milano, Italy; anna.ferretti@scitec.cnr.it
4 Department of Science and Technological Innovation, DISIT, University of Eastern Piedmont, Via T. Michel 11, 15121 Alessandria, Italy; chiara.bisio@uniupo.it
5 Université Clermont Auvergne, Clermont Auvergne INP, CNRS, Institut Pascal, F-63000 Clermont-Ferrand, France
6 Laboratory of Plant Biotechnology Applied to Crop Improvement, Faculty of Science of Sfax, University of Sfax, Sfax 3029, Tunisia; imen.fendri@fss.usf.tn
* Correspondence: philippe.michaud@uca.fr; Tel.: +33-473407425

**Temperature (°C):**	25,0	**Duration Used (s):**	60
**Count Rate (kcps):**	385,1	**Measurement Position (mm):**	0,85
**Cell Description:**	Disposable sizing cuvette	**Attenuator:**	5

			**Size (d.nm):**	**% Number:**	**St Dev (d.nm):**
**Z-Average (d.nm):**	892,9	**Peak 1:**	802,7	13,8	349,5
**PdI:**	0,571	**Peak 2:**	4926	0,0	710,9
**Intercept:**	0,847	**Peak 3:**	154,1	86,2	63,95

Result quality : Good

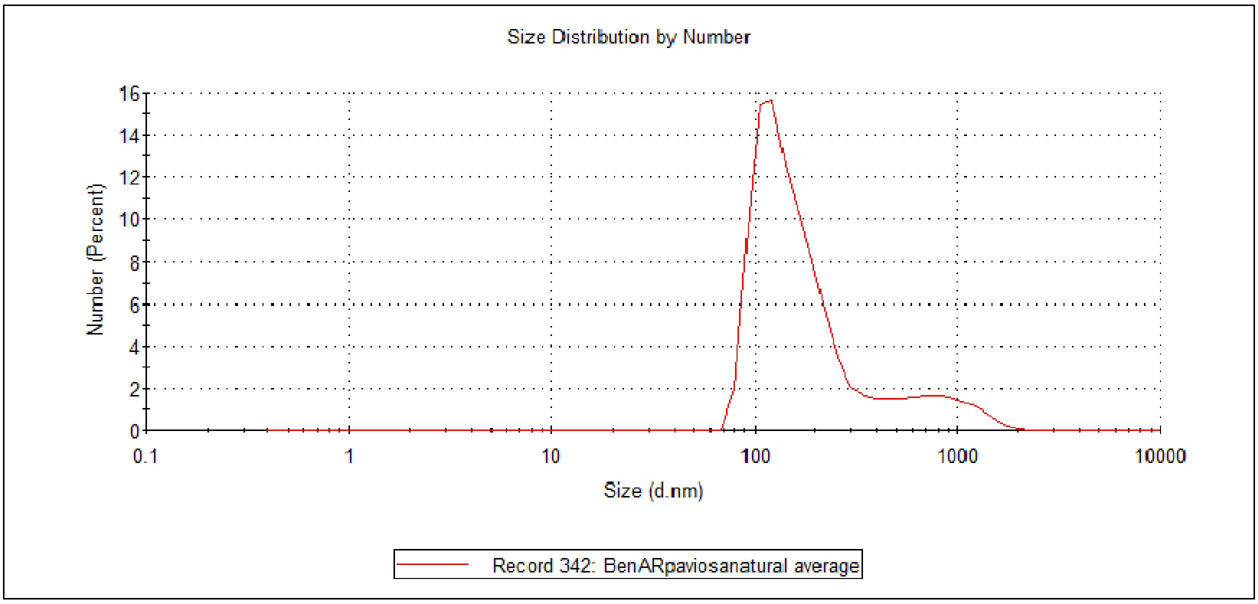

Figure S1. a. DLS analysis for the sample Bentonite.

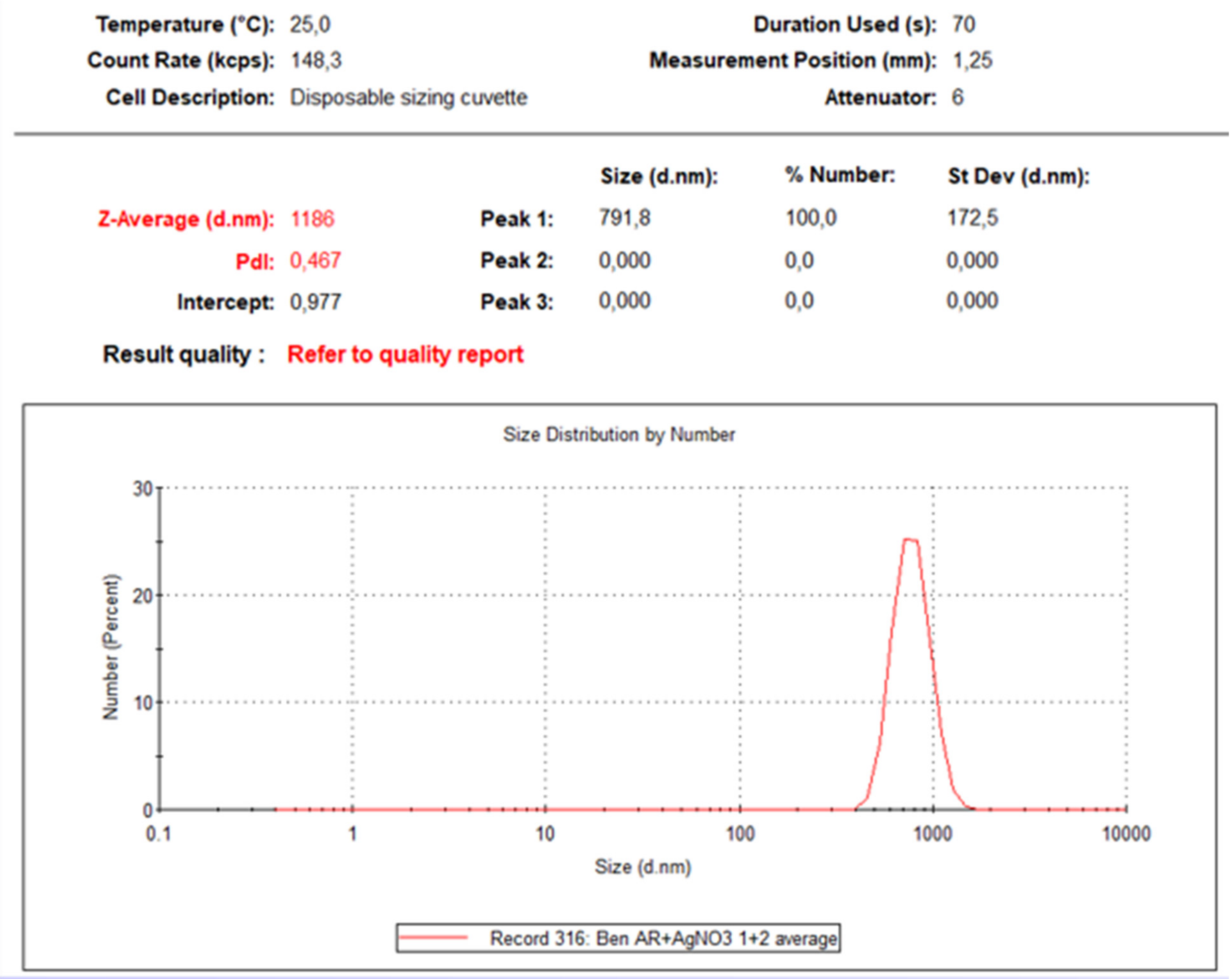

Figure S1. b. DLS analysis for the sample Bentonite-Ag.

**Temperature (°C):**	25,1	**Duration Used (s):**	60
**Count Rate (kcps):**	260,7	**Measurement Position (mm):**	0,65
**Cell Description:**	Disposable sizing cuvette	**Attenuator:**	3

			**Size (d.nm):**	**% Number:**	**St Dev (d.nm):**
**Z-Average (d.nm):**	1074	**Peak 1:**	160,9	88,7	45,65
**PdI:**	0,695	**Peak 2:**	1030	11,3	295,9
**Intercept:**	0,807	**Peak 3:**	0,000	0,0	0,000

Result quality : **Refer to quality report**

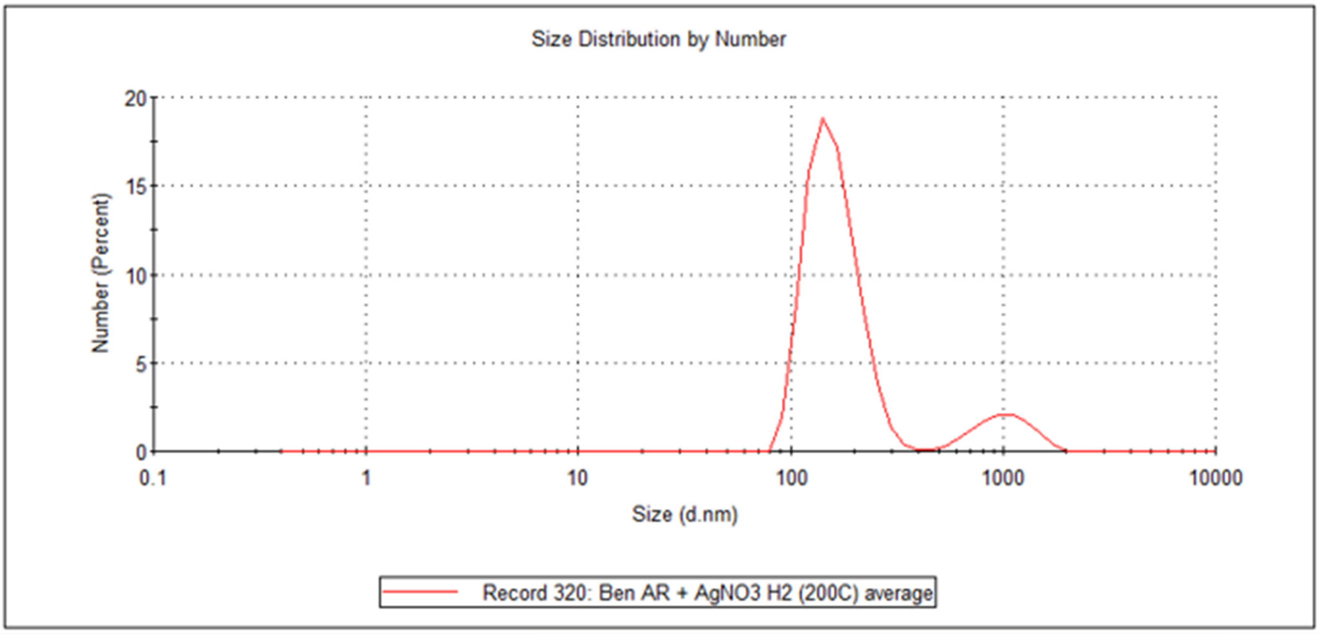

Figure S1. c. DLS analysis for the sample Bentonite-Ag (H_2).
